# Structures of the substrate-binding protein YfeA in apo and zinc-reconstituted holo forms

**DOI:** 10.1107/S2059798319010866

**Published:** 2019-08-22

**Authors:** Christopher D. Radka, Shaunivan L. Labiuk, Lawrence J. DeLucas, Stephen G. Aller

**Affiliations:** a Department of Infectious Diseases, St Jude Children’s Research Hospital, Memphis, TN 38105, USA; b Canadian Macromolecular Crystallography Facility, Canadian Light Source, Saskatoon, SK S7N 2V3, Canada; c Division of Human Exploration and Spaceflight, Aerospace Corporation, El Segundo, CA 90245, USA; d Department of Pharmacology and Toxicology, Birmingham, AL 35294, USA

**Keywords:** substrate-binding protein, cluster A-I, YfeA, *Yersinia pestis*, substrate transfer, X-ray crystallography

## Abstract

In the structural biology of bacterial substrate-binding proteins (SBPs), a growing number of comparisons between substrate-bound and substrate-free forms of metal atom-binding (cluster A-I) SBPs have revealed minimal structural differences between forms. These observations contrast with SBPs that bind substrates such as amino acids or nucleic acids and may undergo >60° rigid-body rotations. Substrate transfer in these SBPs is described by a Venus flytrap model, although this model may not apply to all SBPs. In this report, structures are presented of substrate-free (apo) and reconstituted substrate-bound (holo) YfeA, a polyspecific cluster A-I SBP from *Yersinia pestis*. It is demonstrated that an apo cluster A-I SBP can be purified by fractionation when co-expressed with its cognate transporter, adding an alternative strategy to the mutagenesis or biochemical treatment used to generate other apo cluster A-I SBPs. The apo YfeA structure contains 111 disordered protein atoms in a mobile helix located in the flexible carboxy-terminal lobe. Metal binding triggers a 15-fold reduction in the solvent-accessible surface area of the metal-binding site and reordering of the 111 protein atoms in the mobile helix. The flexible lobe undergoes a 13.6° rigid-body rotation that is driven by a spring-hammer metal-binding mechanism. This asymmetric rigid-body rotation may be unique to metal atom-binding SBPs (*i.e.* clusters A-I, A-II and D-IV).

## Abstract 2

A cluster A-I substrate-binding protein reveals conformational changes, including an asymmetric rigid-body rotation of the flexible lobe, the reordering of a mobile helix and a spring-hammer mechanism.

## 1. Introduction   

Bacterial substrate-binding proteins (SBPs) belong to the widespread ABC superfamily and specifically have a role in sequestering various metabolites, including amino acids, nucleic acids, carbohydrates and metals (Scheepers *et al.*, 2016 ▸). SBPs are thought to freely diffuse through the periplasm of Gram-negative bacteria, anchor to the cytoplasmic membrane of Gram-positive bacteria as a lipoprotein (Felder *et al.*, 1999 ▸) or be covalently fused to membrane transporters (Gouridis *et al.*, 2015 ▸). SBPs are highly abundant in the cell, and in some cases compose up to 40% of Gram-positive surface lipoproteins (Hutchings *et al.*, 2009 ▸). SBPs and cognate ABC importers present attractive therapeutic targets because they are not found in humans, and infection studies have shown that disrupting these substrate-transfer mechanisms greatly attenuates virulence in animal models (Garmory & Titball, 2004 ▸; Paik *et al.*, 2003 ▸; Janulczyk *et al.*, 2003 ▸; Boyer *et al.*, 2002 ▸; Brown *et al.*, 2001 ▸; Fetherston *et al.*, 1999 ▸; Bearden & Perry, 1999 ▸). SBPs have low primary amino-acid sequence identity, but share high tertiary-structural similarity when comparing SBPs that transfer similar substrates (Scheepers *et al.*, 2016 ▸). Trends in the correlative relationships between structure and substrate have been organized into a cluster system that includes structural details unique to each SBP cluster (Berntsson *et al.*, 2010 ▸). All SBPs follow an evolution­arily conserved c-clamp architecture containing α/β globular lobe domains connected by a backbone and interdomain β-strand hinges. In many cases, c-clamps bind substrates at their arch through a mechanism resembling a Venus flytrap, whereby the lobes of a substrate-free c-clamp freely rotate and then tightly clasp a substrate molecule, trapping it inside (Felder *et al.*, 1999 ▸; Mao *et al.*, 1982 ▸).

Support for the Venus flytrap model has come from structural comparisons between substrate-free apo and substrate-bound holo states of many SBPs. Examples include a 43–64° rigid-body rotation observed between apo and holo ribose-binding RBP (cluster B; Björkman & Mowbray, 1998 ▸), a 48° rigid-body rotation observed between apo and holo glutamine-binding GlnBP (cluster F-IV; Hsiao *et al.*, 1996 ▸) and a 39° rigid-body rotation observed between apo and holo molybdate-binding MaModA (cluster D-III; Chan *et al.*, 2010 ▸). Intriguingly, some apo–holo structural comparisons of SBPs that bind other metal atoms (clusters A-I and D-IV) and metal-chelate complexes (cluster A-II) have shown minor structural changes owing to the length of an α-helical backbone linker domain constraining rigid-body rotation of the lobes (Lawrence *et al.*, 1998 ▸). Examples include a 4° rigid-body rotation between apo and holo zinc-binding TroA (cluster A-I; Lee *et al.*, 2002 ▸), a 5° rigid-body rotation between apo and holo iron-binding SfuA/YfuA (cluster D-IV; Shouldice *et al.*, 2005 ▸) and a 12° rigid-body rotation between apo and holo cyanocobalamin-binding BtuF (cluster A-II; Karpowich *et al.*, 2003 ▸). In the case of cluster A-I SBPs, apo–holo structural comparisons are often challenging because the protein purifies in the holo state and does not readily forfeit its substrate. Strategies to generate apo SBP proteins have included partial denaturation, chelation, changes in pH and molecular-biology modifications such as amino-acid substitution and deletion. Presently, several apo structures have been obtained using these methods (Lee *et al.*, 2002 ▸; Wei *et al.*, 2007 ▸; Yatsunyk *et al.*, 2008 ▸; Abate *et al.*, 2014 ▸). Considering that the Venus flytrap model is not valid in the context of this subgroup of SBPs, partial denaturation and mutagenesis experiments with the cluster A-I SBP PsaA have indicated that a spring-hammer mechanism modulates substrate binding at the arch whereby a retracted ‘spring-loaded’ amino acid in the apo protein springs towards a metal atom to lock it in place (Couñago *et al.*, 2014 ▸). However, any structural changes that may occur in addition to the spring-hammer mechanism during substrate binding in cluster A-I SBPs presently remain unknown.

Recently, we determined the crystal structure of holo YfeA, a *Yersinia pestis* polyspecific cluster A-I SBP (Radka, DeLucas *et al.*, 2017 ▸), and reported a method for purifying apo YfeA in the presence of the Yfe transporter by fractionation (Radka *et al.*, 2018 ▸), adding an additional strategy for the production of apo SBP proteins. Analysis of YfeA in the STRING database of known and predicted protein–protein interactions (Szklarczyk *et al.*, 2019 ▸) yields a protein–protein interaction network that includes the Yfe transporter as well as hypothetical interactions with common transporters (*i.e.* MntH and Znu) found in other bacteria. The purification of recombinant YfeA from *Escherichia coli* cells not expressing the Yfe transporter results in the production of holo YfeA, indicating that cross-reactivity between YfeA and other metal-transport systems is unlikely. In this report, we solved the crystal structure of apo YfeA as well as that of holo YfeA that was reconstituted by soaking crystals of apo YfeA with zinc. Comparison of the apo and reconstituted holo YfeA structures shows that YfeA uses a spring-hammer mechanism like that of PsaA to bind metal atoms. A molecular-dynamics study of the flexibility of apo PsaA suggests that metal-free PsaA samples structurally distinct conformations that are not captured by the crystal structure and that these conformations create a larger, solvent-exposed metal-binding site (Deplazes *et al.*, 2015 ▸). The apo YfeA structure enables the visualization of this flexible conformation by crystallography for the first time, as well as calculation of the rigid-body motion that occurs by the spring-hammer mechanism. Apo YfeA contains 111 disordered protein atoms in a mobile helix and a 94.0 Å^2^ metal-binding site. The spring-hammer mechanism triggers ordering of the mobile helix and a reduction of the solvent-accessible metal atom-binding site to 6.1 Å^2^ as the carboxy-terminal lobe undergoes a 13.6° rigid-body rotation in the reconstituted holo structure. The asymmetric rigid-body rotation observed in YfeA is distinct from the Venus flytrap, in which both lobes may rotate.

## 2. Methods   

### 2.1. Cloning, overexpression, purification and crystallization of YfeA   

The cloning, overexpression and purification of native YfeA were identical to methods described previously using the pYFE3 plasmid (Radka, DeLucas *et al.*, 2017 ▸). A key detail of the fractionation protocol is to use gentle methods (*i.e.* pipette aspiration) when resuspending the cells in hypertonic buffer (0.2 *M* Tris pH 8.0, 0.4 *M* NaCl, 2 m*M* EDTA) and hypotonic osmotic lysis buffer (10 m*M* Tris pH 8.0). Methods such as vortex mixing may be too harsh on the spheroplasts, as we have obtained variable results in the overall protein quality and fraction contamination when using vortex mixing. Purified apo native YfeA (18 ± 3 mg ml^−1^) crystallized by the hanging-drop and sitting-drop vapor-diffusion methods at 293 K in the same crystallization condition [20 m*M* bis-Tris pH 6.3, 50 m*M* NaCl, 0.05%(*w*/*v*) NaN_3_, 30%(*w*/*v*) PEG 4000] as holo YfeA-His_10_. Holo YfeA-His_10_ was co-incubated with 2 m*M* EDTA as described previously (Radka, DeLucas *et al.*, 2017 ▸) for energy-dispersive X-ray spectroscopic (EDS) data collection. Partial denaturation experiments utilized purified YfeA-His_10_ protein that was mixed with EDTA to give final concentrations of 5 mg ml^−1^ YfeA and 2 m*M* EDTA. In separate experiments, mixtures were heated by between 25 and 50°C in 5°C increments for 30 s and then cooled for 30 min at 4°C. Precipitate was removed by centrifugation at 20 000*g* for 5 min and the mixture was concentrated to 18 ± 3 mg ml^−1^ YfeA as determined by *A*_280_ and an extinction coefficient of 43 890 *M* ^−1^ cm^−1^ as estimated by *ProtParam* (https://web.expasy.org/protparam). Mixtures were then crystallized in 20 m*M* bis-Tris pH 6.3, 50 m*M* NaCl, 0.05%(*w*/*v*) NaN_3_, 30%(*w*/*v*) PEG 4000. Densitometry calculations were performed using *Licor Image Studio Lite* (http://www.licor.com/bio/products/software/image_studio_lite/).

### 2.2. Reconstitution of holo native YfeA   

Crystals containing apo native YfeA (confirmed by X-ray diffraction) cooled in liquid N_2_ were thawed and subsequently soaked in 4 µl 30%(*w*/*v*) PEG 4000, 20 m*M* bis-Tris pH 6.3, 50 m*M* NaCl, 20 m*M* ZnCl_2_ for 5 min at ambient temperature. After soaking, the crystals were flash-cooled again in liquid N_2_ and X-ray diffraction data were re-collected. The X-ray beam energy for data collection at the Zn *K*-shell electron energy absorption edge was empirically determined by a zinc-edge scan on a control sample containing zinc prior to data collection. All data were collected at the zinc edge after zinc control samples had been used to determine the beam energy.

### 2.3. X-ray data collection, structure solution and refinement   

Diffraction data were collected at 100 K on the Canadian Macromolecular Crystallography Facility (CMCF) 08ID-1 beamline at the Canadian Light Source (University of Saskatchewan). The data-collection strategy was determined using the *iMosflm* strategy function, targeting ≥95% completeness for anomalous X-ray diffraction data. The data were merged and scaled using *HKL*-2000 (Otwinowski & Minor, 1997 ▸). A data completeness of ≥90% and a CC_1/2_ of ≥0.5 in the highest resolution shell were used to determine the resolution limit. CC_anom_ was calculated using *AIMLESS* from the *CCP*4 program suite (Evans & Murshudov, 2013 ▸). Phases were determined by molecular replacement using PDB entry 5uxs (Radka, DeLucas *et al.*, 2017 ▸) as the search model and *Phaser* as implemented in the *PHENIX* suite (Adams *et al.*, 2010 ▸). Model building and refinement were performed using *AutoBuild* in *PHENIX*. Anomalous difference Fourier electron-density peak heights were reported as the maximum density contour (contour ceiling) reported when opening each anomalous map in *UCSF Chimera* (Pettersen *et al.*, 2004 ▸). The figures were generated using *PyMOL* (http://www.pymol.org).

### 2.4. Calculation of rigid-body rotation, site 1 pocket solvent-accessible surface area and randomization of *B* factors   

The YfeA site 1 amino-acid residues (His76, His171, Glu207 and Asp282) were solely selected to define the site 1 pocket. Structure models for each conformational state were loaded into *PyMOL* and site 1 residues were manually selected and defined as a single unique object for solvent-accessible surface-area calculations. Additional parameters were defined (dot_density, 4; dot_solvent, 1) and the zinc ion and water molecules were removed prior to performing calculations using the *PyMOL* get_area command (*i.e.* get_area pocket). Rigid-body rotation and closure percentages were calculated by uploading the atomic coordinates of apo YfeA and reconstituted holo YfeA to the *DynDom* Protein Domain Motion Analysis server (Taylor *et al.*, 2014 ▸; http://fizz.cmp.uea.ac.uk/dyndom/). To test the change in the *B* factor of Glu207 C^δ^ across data sets, the *phenix.model* tool within the *PHENIX* GUI was used to manually set the *B* factors for all atoms to an artificially high value of 100.0 Å^2^. The same tool was then used to randomize the *B* factor of each atom to a value of between 90 and 110 Å^2^. This input file was then modified as three separate input files for refinement with the occupancy of the single Zn atom set to either 0.1, 0.5 or 0.99. The three files were then refined against the data sets.

## 3. Results   

### 3.1. Apo YfeA can be purified from the periplasm of *E. coli* cells expressing the Yfe transporter   

**Figure 1:**
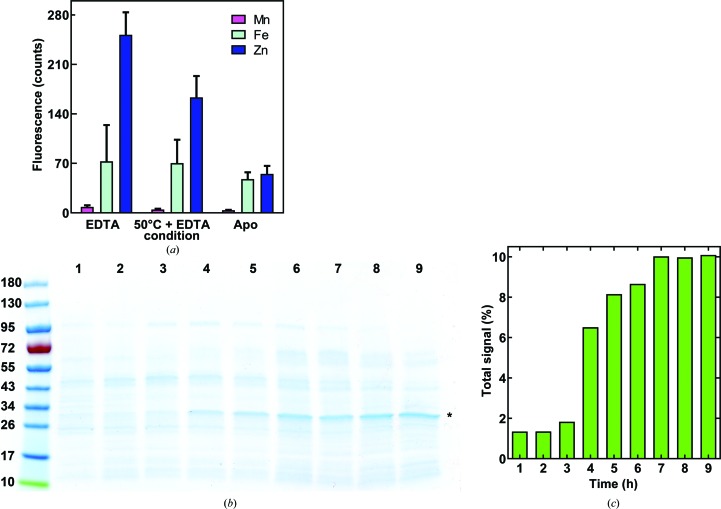
Analysis of YfeA from the periplasm of *E. coli* cells expressing the Yfe transporter. (*a*) EDS spectra of YfeA crystals grown with EDTA in the crystallization buffer (EDTA) and after 50°C partial denaturation in the presence of EDTA with EDTA in the crystallization buffer (50°C + EDTA), and of purified YfeA from the periplasm of *E. coli* cells expressing the Yfe transporter in minimal medium (Apo). Data are represented as the mean of three data sets, with bars indicating the standard error of the mean. EDTA: YfeA from LB co-incubated with 2 m*M* EDTA during crystallization. 50°C + EDTA: 30 s incubation of YfeA with 2 m*M* EDTA at 50°C. Apo: optimal fractionated YfeA overexpressed by pYFE3 autoinduction in cells grown in M9 minimal medium. (*b*, *c*) Analysis of the periplasmic fraction from *E. coli* cells expressing the Yfe transporter. (*b*) SDS–PAGE showing 9 h time-course fractionation after inoculating cells into M9 minimal medium. A representative gel is shown; the experiment was performed three times. An asterisk denotes the electrophoretic mobility position to probe for YfeA. (*c*) Densitometry calculation for the relative abundance of YfeA in the periplasm. Relative abundance is expressed as the percentage of YfeA signal relative to the total periplasm signal in each lane.

**Figure 2:**
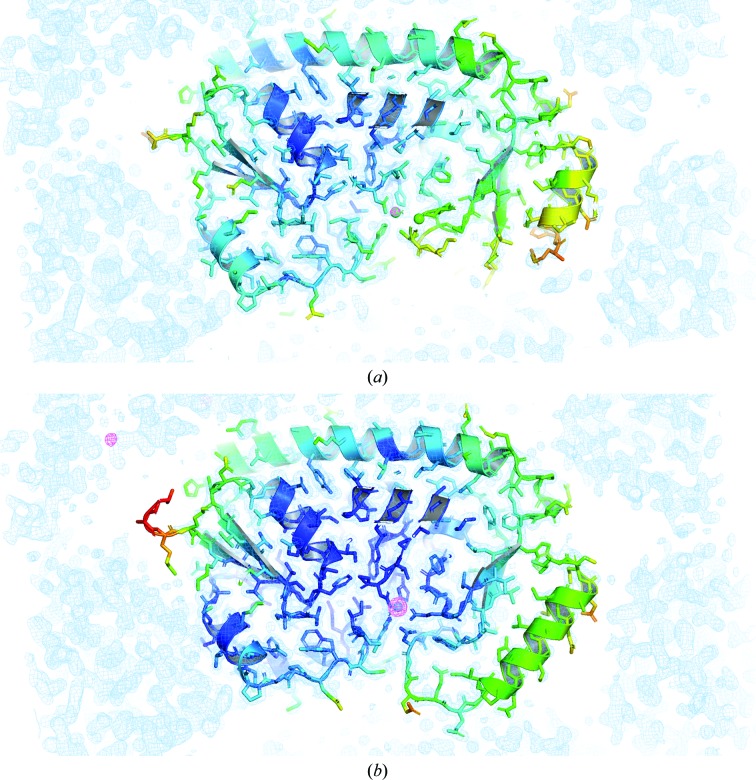
YfeA model fit. (*a*) Apo YfeA. The model, colored by *B* factor, is overlaid with anomalous difference electron density contoured at 5σ (magenta mesh) and electron density calculated from a 2*F*_o_ − *F*_c_ map contoured at 1σ (blue mesh). Missing or broken electron density is apparent for helix 7. (*b*) Reconstituted holo YfeA. The model, colored by *B* factor, is overlaid with anomalous difference electron density contoured at 5σ (magenta mesh) and electron density calculated from a 2*F*_o_ − *F*_c_ map contoured at 1σ (blue mesh). Contiguous electron density is apparent for helix 7.

According to energy-dispersive X-ray spectroscopic (EDS) data and X-ray anomalous difference electron density, zinc is the predominant YfeA substrate at the c-clamp arch, which is referred to as site 1 to distinguish this site from other YfeA metal-binding sites (Radka, DeLucas *et al.*, 2017 ▸). Partial denaturation and chelation conditions marginally reduced the zinc EDS signals to below previously reported levels and did not yield the apo protein by crystallography, despite heating YfeA to 50°C in the presence of EDTA for 10 min [Fig. 1 ▸(*a*)]. Our original fractionation procedure to extract YfeA from the periplasm of *E. coli* cells expressing the full Yfe transporter (Radka, DeLucas *et al.*, 2017 ▸) was designed to identify the substrate at site 1 when YfeA is in the presence of the Yfe transporter in a quasi-native environment. The quality of the fractionation procedure, as measured by the decreased EDS signal in purified YfeA, was improved by using gentle pipetting aspiration specifically when resuspending cells in the hypotonic osmotic lysis buffer (Radka *et al.*, 2018 ▸). Originally, vortex mixing had been used. A possible explanation for the improvement might be that gentle pipetting better preserved the sphero­plasts, reducing inadvertent rupture of the inner membrane and contamination of the periplasm with cytoplasmic contents, including metals that can reintegrate with a potentially apo YfeA (Radka *et al.*, 2018 ▸). These changes enabled the purification and crystallization of seemingly apo YfeA protein exhibiting relatively low EDS signals [Fig. 1 ▸(*a*)]. Considering that EDS is a sensitive technique that can detect metals in trace amounts, X-ray diffraction data were collected at the Zn *K*-shell electron energy absorption edge to determine whether any ordered zinc atoms could be detected in the data. The anomalous difference electron-density map revealed a relatively weak 5.6σ peak height at site 1 [Figs. 2 ▸(*a*) and 3 ▸(*a*)], which is essentially apo considering the anomalous difference peak height at site 1 increases nearly 12-fold to 66.6σ after reconstituting holo YfeA from apo YfeA [Figs. 2( ▸ *b*) and 3 ▸(*b*)]. The weak 5.6σ anomalous difference peak height obtained from the apo data is likely to be caused by a minor contaminant of holo YfeA molecules in the apo crystal. Considering that previous functional assays have shown that the Yfe transporter is active in *E. coli* when overexpressed by the pYFE3 plasmid (Radka *et al.*, 2018 ▸; Bearden *et al.*, 1998 ▸), this result indicates that YfeA–Yfe transporter interactions facilitated substrate transfer *in vivo* and produced apo YfeA protein, as expression of YfeA protein in the absence of the Yfe transporter is always in the holo form and there is no evidence of cross-talk of YfeA with another transporter.

Overexpression of recombinant YfeA and Yfe transporter by the pYFE3 plasmid is driven by the endogenous *Y. pestis yfe* promoter (Bearden *et al.*, 1998 ▸). Transcription of the polycistronic *yfeABCDE* message is upregulated during nutrient-limited conditions, but the relative rates or amounts of recombinant protein made by this method have not been characterized. To better understand gene expression from the *Y. pestis yfe* promoter in the context of the production of sufficient recombinant protein for structural studies, we compared fractionated periplasm aliquots by SDS–PAGE taken hourly up to 9 h post-culture inoculation [Fig. 1 ▸(*b*)]. Densitometry analysis of the SDS–PAGE gel calculated that the YfeA band, previously determined by mass-spectrometric analysis (Radka, DeLucas *et al.*, 2017 ▸), accounted for approximately 6.5% of the total signal in the 4 h time-point lane [Fig. 1 ▸(*c*)]. This value increased to approximately 10% of the total signal in the 7 h time-point lane and remained at 10% through the 9 h time point. Interestingly, by 5 h the YfeA band produced a stronger signal than any other individual band in the lane, and by 7 h YfeA appeared to be the dominant species in the periplasmic fraction by SDS–PAGE. The final yield of purified apo YfeA made using the endogenous *yfe* promoter was approximately 2–3 mg per litre of culture.

This strategy for the purification of apo SBP protein could be applied to other cluster A-I SBPs, although care may need to be taken to optimize expression levels. Some endogenous promoters such as the *yiu* promoter may not drive sufficient gene expression to meet the demands of this experiment (Radka, Chen *et al.*, 2017 ▸).

### 3.2. Apo YfeA contains a disordered flexible lobe   

To preserve the gene spacing in the *yfe* operon, we did not add additional artificial residues for purification. Fortunately, the Fur-induced YfeA, termed native YfeA, crystallized in the same conditions as previously described for His-tagged holo YfeA (Radka, DeLucas *et al.*, 2017 ▸). Crystals of apo native YfeA appeared as thin plates and did not resemble the fine prisms of His-tagged holo YfeA. Some crystals appeared to be jagged, but *phenix.xtriage* analysis of the X-ray diffraction data indicated that the data were not twinned. Diffraction-quality apo native YfeA crystals were observed to crystallize in the orthorhombic space group *P*2_1_2_1_2_1_ and had unit-cell parameters *a* = 40, *b* = 52, *c* = 113 Å, which are 2 Å smaller in the *a* dimension than the unit-cell parameters of the His-tagged holo YfeA crystal form 1 (*a* = 42, *b* = 52, *c* = 113 Å; Radka, DeLucas *et al.*, 2017 ▸). The structure was solved by molecular replacement using the structure of His-tagged holo YfeA crystal form 1 (PDB entry 5uxs). 70% of all residues were built using *AutoBuild* in *PHENIX*, but the remaining residues required manual building because of significant conformational changes in the carboxy-terminal lobe and broken electron density calculated from a 2*F*_o_ − *F*_c_ map. The final model was refined to an *R*_work_ and *R*_free_ of 0.19 and 0.24, respectively. X-ray data-collection and refinement statistics are provided in Table 1 ▸. Intriguingly, the apo data lack electron density calculated from the 2*F*_o_ − *F*_c_ map where the model indicates that residues Pro226–Val238 should be, and there is insufficient electron density in the vicinity to justify modeling these atoms [Figs. 2 ▸(*a*) and 4 ▸(*a*)]. Therefore, only 2042 out of 2153 atoms could be modeled. The missing amino-acid residues constitute the base of the flexible helix 7 and the subsequent loop that have been proposed to undergo structural rearrangement during metal transfer (Radka, DeLucas *et al.*, 2017 ▸). We interpret this observation to suggest the bound-metal-induced structure of the loop may create a high-affinity binding site for the transporter so that the SBP only docks on the transporter when loaded. This same region is predicted to be essential for PsaA recognition by the transmembrane domain of the ABC transporter PsaC (Deplazes *et al.*, 2015 ▸). Future studies include the mutation of Pro226–Val238 to identify key residues of the proposed high-affinity binding site.

### 3.3. YfeA site 1 binds metal by a spring-hammer mechanism as the flexible lobe undergoes structural rearrangement   

**Figure 3:**
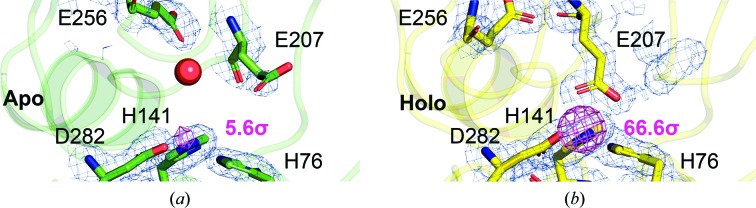
The spring-hammer mechanism in YfeA. The holo YfeA reconstitution experiment reveals zinc binding at site 1 triggers the spring hammer. (*a*, *b*) Enlarged images show the model overlaid with anomalous difference electron density contoured at 5σ (magenta mesh) and electron density calculated from a 2*F*_o_ − *F*_c_ map contoured at 1σ (blue mesh) at site 1. The Zn atom is omitted to emphasize the increase in anomalous difference electron density and peak height (magenta text) after reconstitution of the holo form. A placeholder water molecule (red sphere) is visible coordinating site 1 and the nearby Glu256 in the apo structure (*a*) (green) as site 1 residue Glu207 is disengaged from the other zinc-coordinating residues. Glu207 replaces the placeholder water molecule and engages site 1 in the reconstituted holo structure (*b*) (yellow).

To test the hypothesis that the holo form of YfeA could be reconstituted while YfeA remained crystallized, we thawed the cooled crystals of the apo protein from which we had previously collected X-ray diffraction data to confirm that they were indeed in the apo state. After thawing the crystals to room temperature, we performed a zinc-soaking experiment and re-collected X-ray diffraction data at the Zn *K*-shell electron energy absorption peak. X-ray data-collection and refinement statistics are provided in Table 1 ▸. A 5 min soak produced fully reconstituted holo YfeA, and comparison of the reconstituted holo structure with the apo structure enabled visualization of the structural rearrangement of the mobile helix in the flexible lobe. The reconstituted holo structure shows re-ordering of residues Pro226–Val238, and thus 2153 out of 2153 atoms were modeled [Figs. 2 ▸(*b*), 3 ▸(*b*) and 4 ▸(*b*)]. Amino-acid residues in helices 7 and 8 that are ordered in the apo structure shift downwards and inwards 5 Å towards site 1, closing the c-clamp and reducing solvent access to the site 1 pocket [Figs. 3 ▸(*b*) and 4 ▸(*b*)]. The site 1 solvent-accessible surface area (SASA) calculated by *PyMOL* decreases from 94.0 Å^2^ in the apo structure to 6.1 Å^2^ in the zinc-soaked structure (Table 2 ▸).

**Figure 5:**
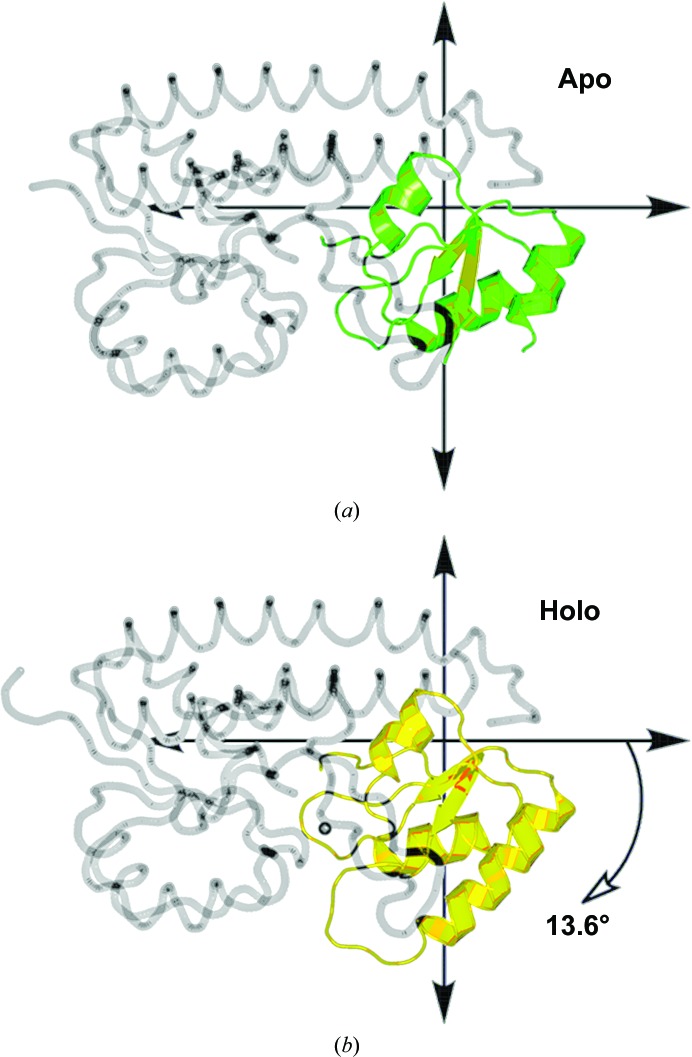
Rigid-body rotation of the YfeA flexible lobe. The *DynDom* Protein Domain Motion Analysis server (Taylor *et al.*, 2014 ▸) calculates a mechanical hinge in the carboxy-terminal domain flexible lobe. The relative position of the hinge axis is at the origin in each coordinate plane along the *z* axis that would project out of the page. Relative to its position in the apo structure (*a*) (green), the flexible lobe undergoes a 13.6° rigid-body rotation in the reconstituted holo structure (*b*) (yellow).

The *DynDom* Protein Domain Motion Analysis server (Taylor *et al.*, 2014 ▸) independently identified a well defined mechanical hinge in the carboxy-terminal lobe. A mechanical hinge creates a stable hinge axis for precise control of domain closure (Hayward, 1999 ▸) and may contain twist or closure axes depending on their direction (Hayward *et al.*, 1997 ▸). The *DynDom* server also calculated a rigid-body rotation of 13.6° and a total closure percentage of 73.7% between the apo and the reconstituted holo structures (Fig. 5 ▸). The closure axis of rotation is located at the origin of the coordinate plane shown in Fig. 5 ▸ along a *z*-axis pointing out of the page. The c-clamp closure percentage and site 1 SASA have an inverse relationship, emphasizing that the reduced access to site 1 is related to the closure motion of the carboxy-terminal domain flexible lobe.

Close inspection of the changes in site 1 between the apo and the reconstituted holo forms identifies the key change that triggers the spontaneous refolding of the mobile helix in the flexible lobe and closure of the c-clamp. In the apo structure, the site 1 residue Glu207 is completely disengaged and a placeholder water molecule is observed to coordinate the nearby residue Glu256 and to establish tetrahedral coordination of a site 1 water molecule [Fig. 3 ▸(*a*)]. In this conformation, the site 1 residues His76, His141 and Asp282 remain grouped together as an electrostatic touch fastener primed to mate with a metal atom. The engagement of the electrostatic touch fastener with a metal atom pulls the β-strand 5 hinge inwards and releases the Glu207 spring-hammer. The firing of the Glu207 spring-hammer triggers the flexible lobe to rotate about the hinge and close the c-clamp. Therefore, YfeA uses the same spring-hammer mechanism to bind metal atoms as described for PsaA (Couñago *et al.*, 2014 ▸). We reason that the Yfe transporter can extract zinc from site 1 and that zinc binding does not irreversibly inactivate YfeA because zinc-free apo YfeA was purified from the zinc-containing *E. coli* periplasm for this study, and zinc binding provokes the Glu207 spring-hammer and triggers closure of the c-clamp.

### 3.4. YfeA transitions from apo to holo in the crystal   

In this crystallographic study, two clear states have been described: apo and reconstituted holo YfeA. A statistical measure that describes the difference between the two states is the atomic *B* factor, and the changes that occur from the spring-hammer mechanism between states influence the site 1 *B* factors. Overall, the site 1 *B* factors in the reconstituted holo data set were 27–53% less than the site 1 *B* factors in the apo data set, and the greatest change occurs in the Glu207 spring-hammer (Table 1 ▸). The Glu207 *R*-group carboxylate carbon (C^δ^), or the head of the hammer, was the focus of this analysis because the position of this carbon changes more than any of the other site 1 atoms after crystal soaking. To control for the minor difference in data-resolution cutoffs (1.76 Å for the apo form and 1.79 Å for the reconstituted holo form), we compared the ratio of the atomic *B* factor to the average atomic *B* factor for each data set. In the apo data, the ratio of the Glu207 C^δ^ atomic *B* factor to the average atomic *B* factor is 1.63, whereas in the reconstituted holo data this ratio is 0.79. As a more robust test for the transition, we randomized the *B* factors of all atoms at artificially high values (90–110 Å^2^) and repeated the model refinement. Under these conditions, the ratios are 1.78 for the apo form and 0.74 for the reconstituted holo form. The calculations from both analyses are in good agreement, illustrating differing degrees of Glu207 hammer-head atomic disorder between distinct states.

Another statistical measure that might be used to describe the difference between the apo and reconstituted holo data is *R*_free_, which will assess the model quality of the conformational changes that occur between states (*i.e.* rigid-body rotation and ordering of the mobile helix). For this analysis, we refined each structure against the electron density from the other data set (Table 2 ▸). The *R*_free_ for the apo structure increases from 0.24 to 0.31 when refined against electron density calculated from the reconstituted holo experiment. The *R*_free_ for the reconstituted holo structure increases from 0.22 to 0.30 when refined against electron density calculated from the apo experiment. This comparison demonstrates that the structures represent distinct states and that the conformational changes that occur between the states are genuine.

## 4. Discussion   

### 4.1. Induced-fit conformational changes in YfeA   

The stable presentation of apo and reconstituted holo YfeA trapped within a crystal lattice allowed the induced-fit conformational changes that occur in response to substrate binding to be detected. In the apo state, YfeA has three grouped (His76/His141/Asp282) site 1 metal-binding residues linked to a rigid amino-terminal domain lobe that serve as an electrostatic attractant for metal ions. A fourth, disengaged site 1 residue (Glu207) is linked to a flexible carboxy-terminal domain lobe containing 111 disordered protein atoms from a mobile helix. The disengaged residue is also a spring-hammer, coordinated by a water molecule to help keep the metal-binding site open with 94 Å^2^ solvent exposure. When a metal ion diffuses to site 1, electrostatic attraction to the three grouped residues causes the metal ion to evict a site 1 placeholder water molecule. The spring-hammer displaces its own coordinating water molecule to engage and lock the metal ion in site 1. This conformational change triggers the mobile helix to reorder as the hammer draws the flexible lobe towards the rigid lobe in an asymmetric 13.6° rigid-body rotation. This asymmetric closure reduces the site 1 solvent exposure to 6.1 Å^2^ and contrasts with the symmetrical rigid-body rotation of the Venus flytrap model that describes other SBPs. We speculate that the asymmetric rigid-body rotation represents the opening of the c-clamp by the ABC transporter and its closing by induced fit.

Intermediate conformational changes that could occur during metal binding might be detectable by varying the crystal-soaking parameters either in time or temperature. It is important to empirically determine the Zn *K*-shell electron energy absorption edge for each synchrotron visit, as the necessary wavelength for X-ray diffraction data collection can slightly change between synchrotron visits. These differences are apparent in Table 1 ▸ (1.28242 Å versus 1.28232 Å). Apo crystals should also be confirmed by X-ray diffraction data collected at the Zn *K*-shell electron energy absorption edge prior to attempting soaking experiments since synchrotron time is limited.

### 4.2. Asymmetric rigid-body rotation in other cluster A-I SBPs   

**Figure 6:**
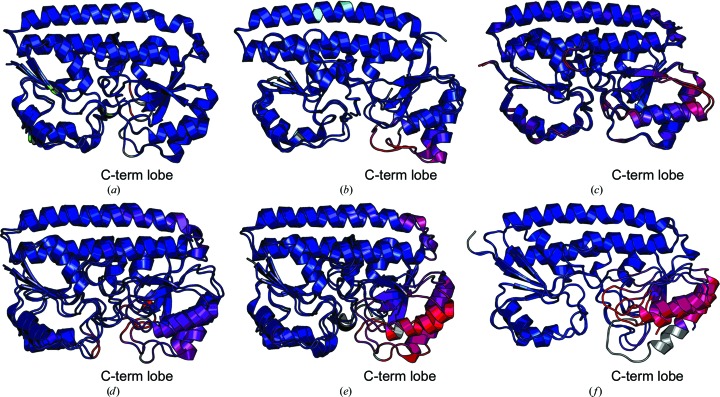
Structural alignment of apo and holo cluster A-I SBPs colored by r.m.s.d. (*a*) SitA, PDB entries 4oxr (apo)/4oxq (holo). (*b*) ZnuA, PDB entries 2ps3 (apo)/2ps0 (holo). (*c*) TroA, PDB entries 1k0f (apo)/1toa (holo). (*d*) PsaA, PDB entries 3zk7 (apo)/3ztt (holo). (*e*) MntC, PDB entries 4nnp (apo)/5hdq (holo). (*f*) YfeA, apo quasi-native YfeA/reconstituted holo quasi-native YfeA. The distances between aligned C^α^ atoms are colored by a spectrum from minimum pairwise r.m.s.d. (blue) to maximum pairwise r.m.s.d. (red). Unaligned C^α^ atoms are colored gray.

Structural comparison of apo and holo forms of cluster A-I SBPs indicate the minimal changes that occur between forms occurs in the carboxy-terminal lobe. Specifically, these changes occur at the base of the carboxy-terminal lobe in proximity to the mobile helix (Fig. 6 ▸). Apo SitA [Fig. 6 ▸(*a*)] was generated by EDTA chelation (Abate *et al.*, 2014 ▸), apo ZnuA [Fig. 6 ▸(*b*)] was generated by partial denaturation and EDTA chelation (Yatsunyk *et al.*, 2008 ▸; Wei *et al.*, 2007 ▸), apo TroA [Fig. 6 ▸(*c*)] was generated by partial denaturation and phenanthroline chelation (Lee *et al.*, 2002 ▸), apo PsaA [Fig. 6 ▸(*d*)] was generated by partial denaturation and EDTA chelation (Couñago *et al.*, 2014 ▸), apo MntC [Fig. 6 ▸(*e*)] was generated by monoclonal antibody interaction (Ahuja *et al.*, 2015 ▸) and apo YfeA [Fig. 6 ▸(*f*)] was generated by cell fractionation after interaction with the Yfe transporter. The holo forms of TroA (Lee *et al.*, 1999 ▸) and PsaA (McDevitt *et al.*, 2011 ▸) used in this analysis came from separate reports to their apo counterparts. Structural comparison of apo and holo SitA shows negligible changes between forms, but this may be because the method of generating the apo protein did not include partial denaturation or protein–protein interaction. Conversely, the greatest degree of conformational change between apo and holo forms is observed in the MntC and YfeA comparisons, which both required protein–protein interactions. Related changes in the carboxy-terminal lobe base by apo- and holo-form comparisons support the occurrence of asymmetric rigid-body rotation in other cluster A-I SBPs. The MntC and YfeA comparisons yielding the greatest degree of change suggest that protein–protein interactions may be required to be fully trigger substrate-transfer conformational changes.

### 4.3. Potential SBP–ABC transporter recognition motif   

Owing to their high conservation across bacterial species, SBPs have shown promise as vaccine antigens against Gram-positive organisms, conferring serotype-independent immunity in animal studies (Gonzalez-Miro *et al.*, 2017 ▸; Martin & Mulks, 1999 ▸). SBPs and ABC transporters have also shown promise in biotechnological applications, including microbial diagnostics (McKevitt *et al.*, 2006 ▸; Reynolds *et al.*, 2009 ▸), fluorescent biosensor uptake (Frommer & Deuschle, 2005 ▸) and increased nutrient uptake (Turano *et al.*, 2015 ▸). Although SBPs share a common c-clamp and high structural similarity with SBPs that bind similar substrates (Radka, Chen *et al.*, 2017 ▸), SBPs are generally not interchangeable, with a few exceptions of SBP cross-talk with noncognate ABC transporters (Létoffé *et al.*, 2006 ▸; Park *et al.*, 1998 ▸). Should an SBP be designed for cross-talk with a noncognate ABC transporter, applications could be conceived such as a mutant SBP designed to deliver an inhibitor to an ABC transporter, or a mutant SBP designed to deliver a new substrate to a highly abundant ABC transporter. A recent study reporting the apo structure of the cluster A-I SBP MntC used a monoclonal antibody to trap the apo form (Ahuja *et al.*, 2015 ▸). This antibody recognizes the MntC equivalent of the YfeA helix 7. We speculate that the structural equivalents of YfeA mobile helix 7 and adjoining loops may contain the key site of interaction between SBP and ABC transporter necessary for instigating the asymmetric rigid-body rotation. More importantly, this motif may contain a recognition sequence that communicates a cognate SBP–ABC transporter interaction, as proposed for PsaA (Deplazes *et al.*, 2015 ▸). Mutating the structural equivalent of helix 7 and the subsequent loop to explore loss of function (no substrate transfer) and gain of function (cross-talk) may reveal short polypeptide sequences that could be synthesized to mimic the recognition motif and potentially inhibit a transporter of interest.

## Supplementary Material

PDB reference: apo YfeA, 6q1c

PDB reference: reconstituted holo YfeA, 6q1d

## Figures and Tables

**Figure 4:**
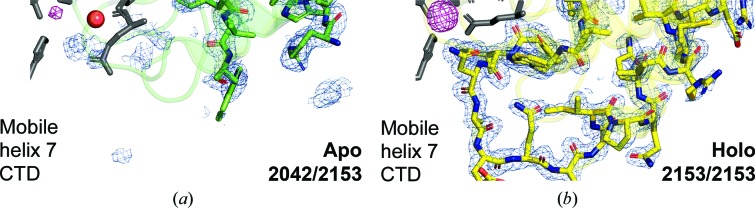
Atomic ordering of the YfeA flexible lobe. The holo YfeA reconstitution experiment reveals atomic ordering of flexible lobe helix 7. (*a*, *b*) Enlarged images showing the model overlaid with anomalous difference electron density contoured at 5σ (magenta mesh) and electron density calculated from a 2*F*_o_ − *F*_c_ map contoured at 1σ (blue mesh). The Zn atom is omitted to emphasize the increase in anomalous difference electron density across soaks, and the site 1 placeholder water molecule (red sphere) and residues (gray sticks) are shown. In the apo structure (*a*) (green), 2042 out of 2153 protein atoms are modeled and there is a visible gap between two flexible lobe residues where Pro226–Val238 should be. In the reconstituted holo structure (*b*) (yellow), with the spring-hammer having engaged the Zn atom, all amino acids are resolved as 2153 out of 2153 protein atoms are modeled.

**Table 1:** Data-collection and refinement statistics Values in parentheses are for the highest resolution shell.

Crystal	Apo YfeA	Reconstituted holo YfeA
PDB code	6q1c	6q1d
Data collection		
Wavelength (Å)	1.28242	1.28232
Space group	*P*2_1_2_1_2_1_	*P*2_1_2_1_2_1_
*a*, *b*, *c* (Å)	40.0, 52.4, 113.3	39.9, 50.9, 115.5
Resolution (Å)	50.0–1.76 (1.79–1.76)	50.0–1.79 (1.82–1.79)
Unique reflections	24085 (1082)	23030 (1135)
Completeness (%)	98.5 (90.5)	99.9 (99.9)
Multiplicity	3.5 (2.5)	6.7 (6.3)
CC_1/2_	92.3 (75.4)	96.7 (97.3)
Overall CC_anom_ †	4.0	36.9
*R*_merge_ (%)	8.5 (38.2)	8.9 (42.8)
*R*_meas_ (%)	9.9 (48.0)	9.7 (46.8)
*R*_p.i.m._ (%)	5.0 (28.5)	3.7 (18.7)
Mean *I*/σ(*I*)	35.4 (2.0)	43.1 (4.6)
Refinement		
Resolution (Å)	38.5–1.76 (1.78–1.76)	46.6–1.79 (1.81–1.79)
No. of non-anomalous reflections	24035	22885
Completeness (%)	94.6 (75.6)	99.8 (98.7)
*R*_work_ (%)	19.0 (28.6)	18.4 (25.3)
*R*_free_ ‡ (%)	23.9 (32.9)	21.9 (26.8)
Wilson *B* factor (Å^2^)	30.7	26.3
Average *B* factors (Å^2^)		
Overall	35.9	31.2
Protein atoms	2042	2153
Solvent atoms	166 H_2_O	158 H_2_O, 1 Zn
No. of molecules in ASU§	1	1
R.m.s.d., bonds (Å)	0.007	0.007
R.m.s.d., angles (°)	0.776	0.815
Ramachandran plot		
Favored (%)	99.6	97.1
Allowed (%)	0.39	2.94
Outliers (%)	0	0
Clashscore	4.41	1.16
*MolProbity* score	1.22	0.99
Model parameters		
Average *B* factors (Å^2^)		
His76	34.6	25.1
His141	27.5	20.2
Glu207	50.0	23.4
Asp282	31.9	19.5
Zinc	Not modeled	27.2
Occupancy		
Zinc	Not modeled	0.42
Anomalous difference (σ)		
Zinc	5.6	66.6

† Calculated by *AIMLESS* from the *CCP*4 suite.

‡ The test set uses ∼5% of data.

§ ASU, asymmetric unit.

**Table 2:** Structure-model comparisons

Structure	*R*_free_ (apo data)	*R*_free_ (holo data)	Site 1 SASA (Å^2^)	Glu207 C^δ^ *B* (atom/average)	Glu207 C^δ^ *B* randomized (atom/average)
Apo	0.24	0.31	94.0	1.63	1.78
Holo	0.30	0.22	6.1	0.79	0.74

## References

[bb1] Abate, F., Malito, E., Cozzi, R., Lo Surdo, P., Maione, D. & Bottomley, M. J. (2014). *Biosci. Rep.* **34**, e00154. 10.1042/BSR20140088 PMC4242081 25311310

[bb2] Adams, P. D., Afonine, P. V., Bunkóczi, G., Chen, V. B., Davis, I. W., Echols, N., Headd, J. J., Hung, L.-W., Kapral, G. J., Grosse-Kunstleve, R. W., McCoy, A. J., Moriarty, N. W., Oeffner, R., Read, R. J., Richardson, D. C., Richardson, J. S., Terwilliger, T. C. & Zwart, P. H. (2010). *Acta Cryst.* D**66**, 213–221. 10.1107/S0907444909052925 PMC2815670 20124702

[bb3] Ahuja, S., Rougé, L., Swem, D. L., Sudhamsu, J., Wu, P., Russell, S. J., Alexander, M. K., Tam, C., Nishiyama, M., Starovasnik, M. A. & Koth, C. M. (2015). *Structure*, **23**, 713–723. 10.1016/j.str.2015.01.020 25752540

[bb4] Bearden, S. W. & Perry, R. D. (1999). *Mol. Microbiol.* **32**, 403–414. 10.1046/j.1365-2958.1999.01360.x 10231495

[bb5] Bearden, S. W., Staggs, T. M. & Perry, R. D. (1998). *J. Bacteriol.* **180**, 1135–1147. 10.1128/jb.180.5.1135-1147.1998 PMC107000 9495751

[bb6] Berntsson, R. P., Smits, S. H. J., Schmitt, L., Slotboom, D.-J. & Poolman, B. (2010). *FEBS Lett.* **584**, 2606–2617. 10.1016/j.febslet.2010.04.043 20412802

[bb7] Björkman, A. J. & Mowbray, S. L. (1998). *J. Mol. Biol.* **279**, 651–664. 10.1006/jmbi.1998.1785 9641984

[bb8] Boyer, E., Bergevin, I., Malo, D., Gros, P. & Cellier, M. F. M. (2002). *Infect. Immun.* **70**, 6032–6042. 10.1128/IAI.70.11.6032-6042.2002 PMC130432 12379679

[bb9] Brown, J. S., Gilliland, S. M. & Holden, D. W. (2001). *Mol. Microbiol.* **40**, 572–585. 10.1046/j.1365-2958.2001.02414.x 11359564

[bb10] Chan, S., Giuroiu, I., Chernishof, I., Sawaya, M. R., Chiang, J., Gunsalus, R. P., Arbing, M. A. & Perry, L. J. (2010). *Acta Cryst.* F**66**, 242–250. 10.1107/S1744309109055158 PMC2833028 20208152

[bb11] Couñago, R. M., Ween, M. P., Begg, S. L., Bajaj, M., Zuegg, J., O’Mara, M. L., Cooper, M. A., McEwan, A. G., Paton, J. C., Kobe, B. & McDevitt, C. A. (2014). *Nature Chem. Biol.* **10**, 35–41. 10.1038/nchembio.1382 24212134

[bb12] Deplazes, E., Begg, S. L., van Wonderen, J. H., Campbell, R., Kobe, B., Paton, J. C., MacMillan, F., McDevitt, C. A. & O’Mara, M. L. (2015). *Biophys. Chem.* **207**, 51–60. 10.1016/j.bpc.2015.08.004 26379256

[bb13] Evans, P. R. & Murshudov, G. N. (2013). *Acta Cryst.* D**69**, 1204–1214. 10.1107/S0907444913000061 PMC3689523 23793146

[bb14] Felder, C. B., Graul, R. C., Lee, A. Y., Merkle, H. P. & Sadee, W. (1999). *AAPS PharmSci*, **1**, E2. 10.1208/ps010202 PMC2761117 11741199

[bb15] Fetherston, J. D., Bertolino, V. J. & Perry, R. D. (1999). *Mol. Microbiol.* **32**, 289–299. 10.1046/j.1365-2958.1999.01348.x 10231486

[bb52] Frommer, W. B. & Deuschle, K. (2005). Patent US20100138944A1.

[bb16] Garmory, H. S. & Titball, R. W. (2004). *Infect. Immun.* **72**, 6757–6763. 10.1128/IAI.72.12.6757-6763.2004 PMC529116 15557595

[bb17] Gonzalez-Miro, M., Rodriguez-Noda, L., Farinas-Medina, M., Garcia-Rivera, D., Verez-Bencomo, V. & Rehm, B. H. A. (2017). *Heliyon*, **3**, e00291. 10.1016/j.heliyon.2017.e00291 PMC5390691 28435909

[bb18] Gouridis, G., Schuurman-Wolters, G. K., Ploetz, E., Husada, F., Vietrov, R., de Boer, M., Cordes, T. & Poolman, B. (2015). *Nature Struct. Mol. Biol.* **22**, 57–64. 10.1038/nsmb.2929 25486304

[bb19] Hayward, S. (1999). *Proteins*, **36**, 425–435. 10450084

[bb20] Hayward, S., Kitao, A. & Berendsen, H. J. C. (1997). *Proteins*, **27**, 425–437. 10.1002/(sici)1097-0134(199703)27:3<425::aid-prot10>3.0.co;2-n 9094744

[bb21] Hsiao, C.-D., Sun, Y.-J., Rose, J. & Wang, B.-C. (1996). *J. Mol. Biol.* **262**, 225–242. 10.1006/jmbi.1996.0509 8831790

[bb22] Hutchings, M. I., Palmer, T., Harrington, D. J. & Sutcliffe, I. C. (2009). *Trends Microbiol.* **17**, 13–21. 10.1016/j.tim.2008.10.001 19059780

[bb23] Janulczyk, R., Ricci, S. & Björck, L. (2003). *Infect. Immun.* **71**, 2656–2664. 10.1128/IAI.71.5.2656-2664.2003 PMC153223 12704140

[bb24] Karpowich, N. K., Huang, H. H., Smith, P. C. & Hunt, J. F. (2003). *J. Biol. Chem.* **278**, 8429–8434. 10.1074/jbc.M212239200 12468528

[bb25] Lawrence, M. C., Pilling, P. A., Epa, V. C., Berry, A. M., Ogunniyi, A. D. & Paton, J. C. (1998). *Structure*, **6**, 1553–1561. 10.1016/s0969-2126(98)00153-1 9862808

[bb26] Lee, Y.-H., Deka, R. K., Norgard, M. V., Radolf, J. D. & Hasemann, C. A. (1999). *Nature Struct. Biol.* **6**, 628–633. 10.1038/10677 10404217

[bb27] Lee, Y.-H., Dorwart, M. R., Hazlett, K. R., Deka, R. K., Norgard, M. V., Radolf, J. D. & Hasemann, C. A. (2002). *J. Bacteriol.* **184**, 2300–2304. 10.1128/JB.184.8.2300-2304.2002 PMC134957 11914363

[bb28] Létoffé, S., Delepelaire, P. & Wandersman, C. (2006). *Proc. Natl Acad. Sci. USA*, **103**, 12891–12896. 10.1073/pnas.0605440103 PMC1568943 16905647

[bb29] Mao, B., Pear, M. R., McCammon, J. A. & Quiocho, F. A. (1982). *J. Biol. Chem.* **257**, 1131–1133. 7035444

[bb30] Martin, P. R. & Mulks, M. H. (1999). *FEMS Immunol. Med. Microbiol.* **25**, 245–254. 10.1111/j.1574-695X.1999.tb01349.x 10459579

[bb31] McDevitt, C. A., Ogunniyi, A. D., Valkov, E., Lawrence, M. C., Kobe, B., McEwan, A. G. & Paton, J. C. (2011). *PLoS Pathog.* **7**, e1002357. 10.1371/journal.ppat.1002357 PMC3207923 22072971

[bb50] McKevitt, M., Palzkill, T. & Norris, S. J. (2006). Patent WO2006138324A2.

[bb32] Otwinowski, Z. & Minor, W. (1997). *Methods Enzymol.* **276**, 307–326. 10.1016/S0076-6879(97)76066-X 27754618

[bb33] Paik, S., Brown, A., Munro, C. L., Cornelissen, C. N. & Kitten, T. (2003). *J. Bacteriol.* **185**, 5967–5975. 10.1128/JB.185.20.5967-5975.2003 PMC225050 14526007

[bb34] Park, J. T., Raychaudhuri, D., Li, H., Normark, S. & Mengin-Lecreulx, D. (1998). *J. Bacteriol.* **180**, 1215–1223. 10.1128/jb.180.5.1215-1223.1998 PMC107010 9495761

[bb35] Pettersen, E. F., Goddard, T. D., Huang, C. C., Couch, G. S., Greenblatt, D. M., Meng, E. C. & Ferrin, T. E. (2004). *J. Comput. Chem.* **25**, 1605–1612. 10.1002/jcc.20084 15264254

[bb36] Radka, C. D., Chen, D., DeLucas, L. J. & Aller, S. G. (2017). *Acta Cryst.* D**73**, 921–939. 10.1107/S2059798317015236 PMC5683015 29095164

[bb37] Radka, C. D., DeLucas, L. J., Wilson, L. S., Lawrenz, M. B., Perry, R. D. & Aller, S. G. (2017). *Acta Cryst.* D**73**, 557–572. 10.1107/S2059798317006349 PMC5505154 28695856

[bb38] Radka, C. D., Radford, L. L., Massicano, A. V. F., DeLucas, L. J., Lapi, S. E. & Aller, S. G. (2018). *J. Vis. Exp.*, e57169. 10.3791/57169 PMC5912328 29443084

[bb51] Reynolds, E. C., O’Brien-Simpson, N. M., Veith, P. D. & Dashper, S. G. (2009). Patent WO2010031127A1.

[bb39] Scheepers, G. H., Lycklama a Nijeholt, J. A. & Poolman, B. (2016). *FEBS Lett.* **590**, 4393–4401. 10.1002/1873-3468.12445 27714801

[bb40] Shouldice, S. R., McRee, D. E., Dougan, D. R., Tari, L. W. & Schryvers, A. B. (2005). *J. Biol. Chem.* **280**, 5820–5827. 10.1074/jbc.M411238200 15576371

[bb41] Szklarczyk, D., Gable, A. L., Lyon, D., Junge, A., Wyder, S., Huerta-Cepas, J., Simonovic, M., Doncheva, N. T., Morris, J. H., Bork, P., Jensen, L. J. & Mering, C. V. (2019). *Nucleic Acids Res.* **47**, D607–D613. 10.1093/nar/gky1131 PMC6323986 30476243

[bb42] Taylor, D., Cawley, G. & Hayward, S. (2014). *Bioinformatics*, **30**, 3189–3196. 10.1093/bioinformatics/btu506 PMC4221117 25078396

[bb53] Turano, F. J., Price, M. B. & Turano, K. A. (2015). Patent WO2016057106A1.

[bb43] Wei, B., Randich, A. M., Bhattacharyya-Pakrasi, M., Pakrasi, H. B. & Smith, T. J. (2007). *Biochemistry*, **46**, 8734–8743. 10.1021/bi700763w 17616151

[bb44] Yatsunyk, L. A., Easton, J. A., Kim, L. R., Sugarbaker, S. A., Bennett, B., Breece, R. M., Vorontsov, I. I., Tierney, D. L., Crowder, M. W. & Rosenzweig, A. C. (2008). *J. Biol. Inorg. Chem.* **13**, 271–288. 10.1007/s00775-007-0320-0 PMC2630496 18027003

